# Searching for the Truth: Elemental Analysis–A Powerful but Often Poorly Executed Technique

**DOI:** 10.1021/acscentsci.2c00761

**Published:** 2022-07-06

**Authors:** Stephen Proctor, Sergio Lovera, Anton Tomich, Vincent Lavallo

**Affiliations:** Department of Chemistry, University of California Riverside, Riverside, California 92521, United States

**Figure 1:**
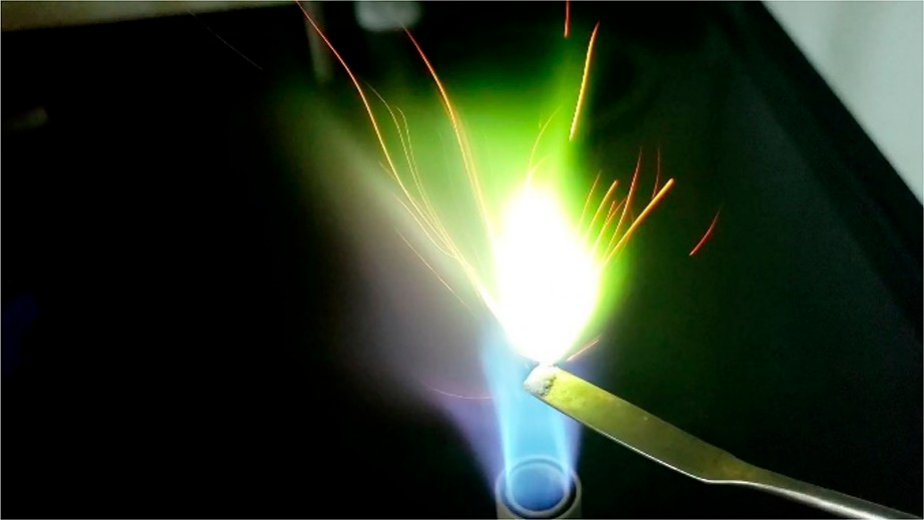
Combustion of carborane salt [Cs^+^][HCB_9_H_9_^–^]. The green-colored emitted light indicates the presence of boron.

Elemental analysis is a broad term utilized to describe multiple techniques to identify the atomic composition of matter. Accurately knowing the composition of a substance is important to understand its properties and demonstrate the homogeneity of a sample. Some of the archaic techniques used by early man and subsequently alchemists involved the combustion of relatively large quantities of material to look for visual cues, such as the emission of specific colors of light, to confirm the presence of elements (Figure 1).

In modern times, technology has advanced to enable more accurate quantification of specific ratios of elements to confirm the atomic composition of a material and demonstrate bulk purity. Of the many known techniques, one of the most pervasive is combustion analysis, where a sample is burned in the presence of an oxygen source, and the volatile gases released are collected and weighed to determine the content of carbon, hydrogen, and nitrogen (CHN analysis). The technique, which was designed by Fritz Pregl (Nobel Prize 1923) to be effective at the microscale (milligrams versus grams), is powerful and very accurate if performed meticulously and correctly.^1^ For most scholarly journals, a requirement of ±0.4% deviation from the theoretical CHN content is required for publication.^2^ Nearly all universities and laboratories rely on external companies to perform this analysis for them as a means of cost savings by avoiding paying a dedicated staff member to run and maintain the instrument. As scientists, we are taught to analyze the raw data or spectra; however, for microanalysis, the raw data are not required for publication, which makes verification from editors and referees impossible, contrary to other characterization requirements (i.e., NMR and X-ray diffraction). Within my research group, several scientists analyze the same raw data, as a matter of routine, prior to submission for publication, but microanalysis companies typically do not provide this raw data, presumably as journals do not require it. This prompts the question: can we trust these values provided without data from a third party, and are the standards set by scholarly journals reasonable and useful?

In an enlightening study^3^ in this issue of *ACS Central Science* titled “An International Study Evaluating Elemental Analysis” led by a multinational cohort of research groups (Kuveeke, Chitnis, Dutton, Martin, and Melen), the efficacy of outsourced CHN analysis was examined. In the study, high-purity compounds were purchased from reputable chemical companies, such as Sigma-Aldrich, and the exact same samples were sent to different laboratories for analysis. They found significant deviations in the results of the CHN content delivered by these companies. The authors also question the standards adopted by various journals, specifically the origin of the ±0.4% rule, and also why that rule is equal for C, H, and N even though they are often found in significantly different weight ratios. It is not clear exactly why there is so much inconsistency between laboratories, but it is likely due to calibration issues of the analytical balances and also human error. The poor execution of these experiments probably has the microanalysis pioneer, Fritz Pregl, turning in his grave! One of the authors had a new instrument in their lab, and it is noteworthy that all analyses from this came back correct, which validates Pregl’s method. This was also the case in a recent study by Kowol and co-workers.^4^

I have been practicing synthetic chemistry for 20 years and have personally experienced or witnessed, as a student/postdoc experimentalist and now as a faculty member evaluating my group and other students, many instances where elemental analysis samples have been sent and resent—sometimes up to 20 times—until the data returned matches the predicted theoretical value within ±0.4%. As a scrutinizing scientist, do you think this is a valid scientific practice? This approach is like playing the lottery. Moreover, companies sometimes offer duplicate and triplicate analyses, but there is no guidance on which value to take, or the average. Fishing for a result that you are expecting and throwing away all of the other data points collected and only reporting the one that matches on a good day does not lead to the truth, and science is the truth. Unfortunately, this is common practice, and I think we need to rethink this approach and the community as a whole needs to reevaluate or adopt different standards. As an author and a reviewer, I also question the language in the journal requirements; for example, Wiley journals state “data should be required to an accuracy within ±0.4%.” The term “should” is ambiguous and does not seem to be enforced equally by reviewers or editors. The reality is, since Pregl’s seminal studies and instrument development, many other techniques have been developed to examine the purity and composition of compounds that complement combustion analysis. I certainly trust my intuition and skills using other techniques (multinuclear NMR, HRMS, single-crystal X-ray diffraction, X-ray photo electron spectroscopy, infrared spectroscopy, etc.) to determine purity with or without CHN combustion analysis, especially as I can assess the raw data from these methods. I feel my expertise combined with my graduate students, whom I carefully select and train, is more trustworthy than a random technician of unknown training running a sample sent in the mail to a far-off location.

To calibrate the reader on the economics, a single sample sent to a lab for analysis costs anywhere from 80 and up to 300 US dollars if it happens to be air sensitive (from my experience). In some synthetic papers, there can be 20–30 new compounds that may require elemental analysis. This certainly leaves a sour taste in my mouth as our tax paying dollars are being spent in a wasteful manner for unreliable or unsubstantiated results. From a psychological standpoint, imagine being a graduate student, particularly a new one, and you find some amazing new chemical entity that passes muster in all respects except for the fact that it is repeatedly returned as a “fail” from a company you do not know. Now you are getting unwanted attention from a stressed-out assistant professor, as we all have been, and your adviser is getting more and more frustrated with you as he needs that paper badly. You have done nothing wrong, and for all we know now your compound could have been perfectly pure, but some unknown technician keeps messing up the combustion analysis, which you are unable to verify. It is not fair to put this burden on our students, as the graduate school and postdoctoral experiences are hard enough. In many cases, such as with a sensitive or fleeting compound, a lack of purity does not diminish the result, but it is required by the journal. A solution to this whole problem would be for each university to have its own dedicated elemental analysis instruments and a highly trained Ph.D. who maintains the facility and trains students on how to properly conduct the experiments and regularly calibrate with appropriate standards. This probably will never happen, as extracting resources from administrators, as one of my colleagues says, is like trying to draw blood from a stone. Alternatively, perhaps we should stop requiring elemental analysis as an essential component of proper chemical characterization. All scientists should be disappointed if editorial boards do not take this work seriously and reconsider their metrics.
